# Movement, touch and talk in Norwegian psychomotor physiotherapy

**DOI:** 10.3389/fresc.2022.1011146

**Published:** 2022-11-17

**Authors:** Eline Thornquist

**Affiliations:** Department of Health and Functioning, Faculty of Health and Social Sciences, Western Norway University of Applied Sciences, Bergen, Norway

**Keywords:** touch, respiration, muscle tension, phenomenology, psychomotor physiotherapy, flexibility

## Abstract

People suffering from musculoskeletal disorders are frequently treated by physiotherapists using a wide variety of therapeutic approaches. In debates over the relative merits of hands-on and hands-off therapies, proponents of hands-off methods have argued that hands-on clinical work turn patients into «passive» recipients. But this is a simplistic proposition. Psychomotor physiotherapy is a Norwegian therapeutic approach characterized by a more or less continuous bodily dialogue combined with talk in examination and treatment sessions alike. Touch is thus a significant part of the ongoing interaction between patient and therapist. Through concrete examples, the article shows how the physiotherapists—using hands-on methods extensively—induce active responses in the patients, aid them to trust their bodies, change dysfunctional habits and become aware of their capacity for handling their health problems. The therapists do this by exploring and molding patients' movements, handling their muscle tensions and encouraging ways of breathing in combination with talk. The examples demonstrate that the body is just as «communicative» as talk itself and how new insights—to patients and therapists alike—can be obtained by juxtaposing verbal and bodily messages systematically. The examples also suggest that the meaning of “touch” varies. Consequently, what touch entails will necessarily inform clinical practice. Combining the theoretical framework of psychomotor therapy with phenomenology, perspectives from neuroscience and insights from the social sciences, the article casts new light on potentials and challenges triggered by the intertwining of talk and bodily handling typical of psychomotor therapy clinical work. A case is made for a view of the body as intrinsically communicative and for a more profound understanding of what not only psychomotor therapy but physiotherapy generally can offer.

## 1. Introduction

Physiotherapists have long debated the relative merits of hands-on and hands-off therapies (1–10). Proponents of hands-off methods have claimed that hands-on clinical work turns patients into passive recipients who leave the responsibility for their health and well-being to health personnel, while active approaches place it with the patients themselves. However, the relationship between approach and effect is not that simple. Physiotherapists commonly point out the benefits of physical activity, but it is quite another matter to champion exercise and training as a panacea for all ailments.

While approaches in physiotherapy vary in the degree to which touch is essential in examination and therapy, I have selected for discussion here Norwegian Psychomotor Physiotherapy (NPMP). In this tradition, a variety of hands-on methods are used in examinations as well as in treatment sessions (11–15).

NPMP was developed jointly by the psychiatrist Trygve Braatøy (1904–1953) and the physiotherapist Aadel Bülow-Hansen (1906–2001). Braatøy had written prolifically prior to and right after World War II about bodily manifestations of psychological problems, while Bülow-Hansen had some 20 years of broad clinical experience. After a chance meeting between the two in 1947 they began a systematic collaboration until Braatøy's untimely death in 1953 (12–15).

Two principles—building the body from the bottom up and treating the whole body regardless of the patient's symptoms and health problems—are based on two fundamental premises: First, that the body is a carrier of the person's past experiences, good and bad, which means that the body “remembers”. Second, that the body is a functional and interacting entity. Disturbances in one part of the body may create imbalances in other parts (12–15).

In NPMP it is recognized that we convey joy and sorrow with our body, and by way of tensions, limited breathing, and guarded movements we constrain ourselves, we curb unpleasantness, despair, and loss. In other words, the body not only *expresses* but also *regulates* feelings. Clinically, the implication is that the entire body must be examined and treated so that the different parts and functions are evaluated in relation to each other. All professional assessments and choices are made based on the dynamic interplay within the body and between the body and emotions (12–15).

Summing up: from the basic tenet in NPMP that feelings and bodily manners are understood as mutually binding and mutually liberating it follows that the body has a function in a psychological and social sense.

The aim of the NPMP *examination* is to *obtain information* about the body's flexibility and versatility. In psychomotor therapy, flexibility entails both the patients' overall mobility and their ability to let themselves *be moved*, whether by another person or simply by gravity. Versatility refers primarily to the person's ability to adapt or adjust respiration, but it includes also the person's capacity for bodily adjustments more generally (concerning tension level, temperature regulation, yielding). The *therapeutic* aim is *change* in the form of a readjustment of the body, based primarily on liberation of tensions and restricted breathing through a broad range of hands-on means, exercises, and movements (12–15).

Psychomotor physiotherapists employ different forms of palpation, massage, manual grips, and other bodily interventions (such as stretching, traction). By assisting and molding patients' movements, inviting their participation, physiotherapists stimulate patients to trust their bodies, change dysfunctional habits, and become aware of their capacity for handling their health problems.

From what has been said so far, it should be clear that NPMP differs from standard physiotherapy in several respects. One more difference should be added: precisely because it takes a long time to change dysfunctional bodily habits, treatment is never rushed. Treatment is usually weekly, sometimes every two or three weeks. Later in the treatment process one session per month or even less is common, and patients are encouraged to do exercises in-between sessions and to explore new ways of moving and acting.

This article seeks to augment current understanding of the significance of touch in physiotherapy by close readings of publications on clinical practice in NPMP drawing on theoretical perspectives from phenomenology and the social sciences as well as recent findings from various fields in the natural sciences.

## 2. Materials and methods

While NPMP as just noted is a practical approach characterized by extended use of hands-on means, published accounts of actual clinical encounters are few and far between. Research on NPMP is dominated by interview studies that focus on patients' and physiotherapists' experience with and opinions on various aspects of the tradition. While such studies may provide important information [see for instance (16)], this domination is problematic because one cannot take it for granted that there is correspondence between what is said *about* practice and what actually takes place (17–19).

The methodical approach adopted here constitutes a theoretically grounded reanalysis of published material on clinical encounters (20–22). Notably, none of these texts were written with the explicit aim to highlight the role of hands-on approaches or the significance of touch more generally. Yet, they demonstrate the ubiquitousness of hands-on methods in NPMP diagnostic encounters as well as in treatment sessions, and how accompanying verbal exchanges between patients and physiotherapists unfold.

Extensive excerpts will show how this combination of touch and talk induce active responses from the patients and contributes to their involvement, participation and learning in the sessions. Deliberately included are passages that demonstrate richness and heterogeneity in the use of touch. They also reveal specific professional challenges in clinical settings.

Ever since NPMP was developed (1947–1953), most psychomotor physiotherapists have worked in the primary health sector. The first case selected for analysis is about the treatment of a patient referred by a physician for “tension headache” because this is a common diagnosis in this part of the health sector. The explicit aim of that article (20) was to highlight, as the subtitle says, “How do we understand patients” tears and how do we react to them?” Motivating the aim was the professional experience of the first author, Fangel, who is the physiotherapist here, with seriously traumatized patients having experienced war and torture.

The second case concerns the treatment of a patient suffering from a serious mental problem and certain bodily dysfunctions. This account (21) was selected because there is increasing public attention to mental health in many parts of the world. There is also growing concern among physiotherapists that psychiatric care has become a rather neglected sector in our profession (6, 23, 24). Literature about NPMP from psychiatry is scarce, and the publication made use of here is the result of a professional interest in NPMP by a group of physicians and psychiatrists (in Tromsø, Norway). The excerpts selected for analysis actualize questions about how movement, touch and hands-on means can be used *and misused*, questions that are highly relevant in our profession. What makes this case especially appropriate is that the patient disliked being touched.

The third and final case is about the NPMP examination, often referred to as a “trial treatment” (22). The patient had several ailments, but he suffered first and foremost from muscle tensions. The case is drawn from my own research, being part of a broader study based on direct observation and video recordings, supplemented by interviews individually of patients and therapists (17). The overall aim was to analyse actual physiotherapy practice in examination settings, making explicit the explanatory models and classificatory schemes underlying this practice. [For further details, see (25)].

Below, particular attention is paid to how touch and hands-on methods are used and how they relate to movement and talk. My comments and analysis will demonstrate how touch, movement and talk are all intrinsically communicative. The text is organized around extensive excerpts from these publications, followed by practice-near and theoretically informed comments and reflections directly related to the descriptive excerpts in each case. This way, the readers are invited to monitor the steps that have been taken in exploring the material at hand.

The three cases and the initial comments will be taken up again in more detail in the Discussion section. There, the aims are twofold: first, to cast new light on the potentials and challenges prompted by the intertwining of talk and bodily handling typical of psychomotor physiotherapy clinical work and second, to clarify central points with immediate relevance to clinical practice in physiotherapy more generally. To this end, the exposition benefits from classical and recent work on phenomenology (26–29), insights from the social sciences (30, 31), as well as new findings in neurology (32, 33) and the neurosciences (34–36).

## 3. Results

### 3.1. Body work and talk—Katrine

Now to the article about how physiotherapists deal with tears in treatment (20). It discusses two cases, one of which is selected. The patient is a 64-year-old woman, Katrine, suffering from tension headache and recurring bouts with migraine—she was “born with a headache”, she jokingly told the physiotherapist [(20), p. 8; all translations to English are mine]. The therapy took place in private practice in the Danish primary health care.

The presentation of the patient starts with a brief reference to the examination and the physiotherapist's assessment, after which a part of the fifth session is described. About the examination the first author, Fangel, concluded that Kathrine “had pronounced bodily control, but also valuable bodily competence” [(20), p. 8]. She was also thought to be “bodily, socially, as well as cognitively resourceful” [(20), p. 8]. On this basis the physiotherapist considered that the patient would benefit from a somewhat invasive loosening treatment.

#### 3.1.1. Fifth session

What follows are excerpts from the article in question (20)—the “I” here is Fangel, the first author:

“We work on her shoulders and chest area while she lies flat on her back. It is difficult for Katrine to release control of her left arm/shoulder and let herself be moved passively. She feels a little uneasy, wondering aloud and slightly annoyed at this: “it is just my arm that you are moving, how hard can it be.” I notice that her breathing is rapid and occurs predominantly in the epigastric region. I put her arm back into place and Katrine sighs lightly. She quiets down immediately, her gaze gets distant, her eyes dim, and she tenses noticeably in her jaw area” [(20), p. 9].

“I remark on what I see and the dimness in her eyes turns into tears as Katrine tells me what she had begun to think about: what it was like when as a child she came home from school. She was afraid of what kind of reception she would get. Together, we discover that Katrine would push her shoulders forward as if to prepare for what she might encounter. Alert at how her mother would be; a mother who was often stern and at times rough” [(20), p. 9].

We are told that this preparedness has been part and parcel of Katrine's being for some 60 years—since a little girl she had armed herself by tensing her muscles—and that when the physiotherapist attempted to loosen Katrine's tension all manner of “images, emotions and words poured forth” [(20), p. 9]. Now, while all of us tense up in certain situations, problems such as dysfunction and pain can arise “if tensions pile up on older tension patterns: Tension and action patterns can come to constitute the basis for other tensions and in time build an entire mesh of bodily constrictions” [(20), p. 9].

As is evident from these excerpts, Fangel is constantly intent on Katrine's bodily responses, interpreting them (“She feels a little uneasy …”), drawing preliminary conclusions, and then letting readers in on how she immediately adjusted her clinical strategy,

“Katrine's associations were a consequence of my touch and what we did with her body. Had I just let her sense her unrest without mentioning what I saw (her eyes dimming and her jaw clenching) she might not have become aware of how the tensions she suffers from today relate to what in the past made her tense up in the first place” [(20), p. 9].

The first author summarizes the session so far by noting that Katrine's newfound insight made it possible for her to address her tensed-up shoulders in a different way,

“Rather than becoming annoyed at being unable to relax she became more alive to how in current situations, too, she readies herself. This made it possible for durable changes to take place” [(20), p. 9].

The authors reflect further on the session by noting the importance of verbalizing experience. But they also alert us to a caveat: physiotherapists are not to function as psychologists. Still, for a patient to put experience into words,

“contributes to an anchoring to reality and to establishing connections. Moreover, if reactions are passed over, they fail to be recognized as real; the dimness in the gaze can be blinked away quickly and the sad feeling can be overheard” [(20), p. 9].

The authors note, however, that to listen attentively is not enough. Being observant is equally important. Thus,

“A physiotherapist, necessarily external to the patient's personal experience, can detect tiny bodily changes such as dim eyes even before the patient is cognizant of it. The therapist can address what has been observed in a respectful manner, after which it is up to the patient whether it becomes a topic in the therapy” [(20), p. 9].

And conversely,

“… to avoid mentioning it is to deprive patients of the possibility of achieving greater self-knowledge and insight into their own reactions” [(20), p. 9].

#### 3.1.2. Comments

Four summary observations follow.

First, the excerpt makes it evident that touch was an integral part of the practice reported on here. Second, it becomes evident, too, that the physiotherapist's attentiveness to the patient's various reactions (her breathing, sighing, gaze and eyes, the changes in muscle tension in other parts of her body) is crucial to how the encounter unfolds therapeutically.

Third, the physiotherapist used massage and various forms of manual grips, aiming to help the patient to arrive at a more appropriate and efficient way of moving. To this end, she explored *different* ways of moving, paying attention to nuances in how the patient experienced the touches and grips.

Fourth, the physiotherapist combined hands-on body work with talk *directly* related to the patient's bodily responses. The point is that this way of going about it is not a first-this, then-something-else approach; it is rather about communication along different channels and levels simultaneously.

In this, the physiotherapist embodies one of the most central tenets in communication theory: you cannot not communicate [(37), p. 48–49]. “Activity and passivity, words and silence all have message value” as Watzlawick et al. express it [(37), p. 49], and as Goffman and other micro-sociologists have stressed is inescapable in any face-to-face situation (31, 38).

Importantly, it also facilitates the patient's active participation in her own healing process, for she is offered the opportunity to gain new and different bodily experiences, something that in itself can boost body awareness and confidence.

In sum, the main message is that bodily ailments can be viewed as the result of adjustments to struggles and strains in life. When a patient, as in Katrine's case, has developed a long-standing state of guarded readiness, it is possible to view it as a functional protection—such as against a stern mother. But this kind of protection is dysfunctional in that the body becomes inappropriately burdened and susceptible to various forms of minor ailments and more debilitating health problems, cf. the NPMP axiom that the body is a functioning and interactive entity.

As emphasized above, a corresponding tenet in NPMP is that the body expresses as well as regulates our emotional life. The reciprocal “slowing down” of body and emotions takes place largely beyond conscious control.

As Braatøy wrote in one of his books:

“Our upbringing or our experiences go much further back than our conscious memory, and this means that our body has assumed habits the origins of which we ourselves cannot remember” [(39), p. 29–30].

It is precisely because as human beings we begin to cultivate bodily habits and ways of being at a very early stage—largely unconsciously, unaware of what we bring with us later in life—that conversations have their limits.

That the body is the centre of experience and a field of expression and thus a historical phenomenon, means that the body is a source of information and knowledge not only for the professional, but also for the patient. However, to make this knowledge of the patient as an embodied social being available requires that we as professionals help the patient to connect the past with the present. This point will be taken up again below.

Now, however, another aspect of the NPMP approach that deserves attention will be examined, namely how bodily proximity and contact can function therapeutically as well as anti-therapeutically. Attempts at treatment can retraumatize by activating past experience and rekindle memories that the patient is unable to handle and process. It is all to do with how treatment is conducted.

### 3.2. Approaching gradually—Anne

The example below concerns a middle-aged woman, Anne, who has been in psychotherapy as well as in psychomotor physiotherapy (21). In focus here are her experience of her body and of the bodily treatment she has received.

Some background information: A general practitioner expressed concern for Anne who had come to him with stomach pains. She suffered from nausea and had become quite emaciated. On top of this she had “strange thoughts” and what she called “odd visions”. The physician recommended a psychiatrist. The psychiatrist concluded that she was psychotic, and she started on psychotherapy and psychomotor physiotherapy in parallel.

What follows are excerpts from a conversation between her and a psychologist shortly after the therapies had taken place.

Anne says in the interview that she had feelings of guilt for everything: “my mom was cross and my dad drank … I was a bed wetter and I was inadequate at everything” [(21), p. 17]. She also said that she had hardly any recollections of her life before she reached 11 or 12 years of age.

In the excerpts below the interviewer (I) is the psychologist. Anne is P (the patient).

“I: I have been told that for many years you have felt nothing, sensed nothing … how was that, didn’t you sense anything from your body?

*P*: It was like being inside a grey cloud. I did what I was told, I replied to what I was asked, besides that there was nothing. It was very strange to discover that I could do what I wanted. My body was a nothing thing. I remember telling X (the psychiatrist) that the body is a fine contraption to stick a head on. Physical education at school repulsed me. I slunk away from these classes.

I: How was it with what others call pain?

*P*: Ah yes, that was weird. You know, out there among women talk often turns to births and it was strange to me because they often talked about how painful it was. I understood that I just had to play along with them because I felt no pain when I gave birth to my boy. I remember the midwife holding her hand on my belly, saying “now you must press, you have pressure contractions”, and I didn't understand because I felt nothing. All this about bodily pain I never felt, not until lately …” [(21), p. 60].

Then the conversation shifts to the physiotherapy treatment:

“I: How did it come about that you went to her [referring to the physiotherapist]?

*P*: First he (the psychiatrist) asked me if I would consider that kind of examination, and I got an appointment … It was really unpleasant to be touched. I didn't like that at all. She (the physiotherapist) said she could see that I was uncomfortable, and I said I could come to her for treatment if she didn't touch me. “We can do exercises”, she said, “to begin with. Then we’ll see how it goes.” I felt relieved at that … Lately she has gradually begun to treat me, first my feet. The first time she just touched them I got nauseous, but now she can touch me everywhere, she can even treat my throat and my face” [(21), p. 60–61].

Later in the interview—and on her own accord—Anne adds that she was given several exercises to do at home and that she “still does them”; she found them useful and kept doing them daily.

### 3.3. Comments

Anne's words illustrate how one's emotional life can be regulated bodily—closing oneself off, making oneself invulnerable, feeling nothing. What she says offers certain pointers to health personnel as regards concrete situations.

Perhaps most fundamentally, one is well advised to be solicitous for patients' experiences while at the same time be clear and unequivocal about one's own assessments and choices.

It appears from what Anne says that the physiotherapist in question expressed what her impressions were: “she said she could see that I was uncomfortable.” Responding like that, the physiotherapist conveys that she has registered the patient's struggle but refrains from evaluating or diagnosing her.

The example suggests a therapeutic practice based on the recognition that the body speaks, that it has a story to tell, that it is not some neutral physical object but a historical phenomenon with its own kind of memory. From this perspective, to touch and handle another person in bodily ways is to approach another person's life history, to encounter that person's good and bad memories.

The case also shows how verbal clarity coupled with bodily restraint and gradual bodily contact (first, activity and exercise, then somatosensory stimulation beginning at the patient's feet, then moving to other body parts) provided a possibility for the patient to establish contact with herself and boost her self-esteem and improve her physical and social function.

It is reasonable to view these effects in connection with Anne being treated by a physiotherapist and a psychiatrist simultaneously. Moreover, she saw both on the same day, always beginning with an hour with the physiotherapist. The thought behind this arrangement was that the bodily approach might activate restrained feelings and submerged conflict material that could be further dealt with in conversation with the psychiatrist.

It is worth pointing out that such collaboration between psychiatrist and physiotherapist was fundamental when Braatøy and Bülow-Hansen began to develop the NPMP approach in a psychiatric setting in the late 1940s. Bülow-Hansen herself put it like this:

“[Braatøy] was interested in two things: one was, what feelings can we *release* through posture and massage, and what can we *use* the physiotherapist for in the psychotherapeutic situation” [(40), p. 9, italics mine].

What becomes clear here, is that Braatøy—by virtue of being a psychiatrist with a psychoanalytic background—was searching for clues to *how bodily approaches could be used in psychiatry*. One might think that Braatøy this way reduced our profession to an implement in his own search for knowledge. But it would be a grave mistake to interpret him as belittling physiotherapy. In fact, it is clear from his copious writings that he had great respect for physiotherapists—especially for Bülow-Hansen.

We should also remember that the two of them were breaking new ground. The entire enterprise comes across as an immense, purposeful, professional experiment. Essentially, it was all about a further development of psychoanalytic therapy.

Elsewhere this has been formulated like this:

“Originally, psychomotor therapy was used by psychiatrist Braatøy *to draw out feelings and conflicts* in persons who resisted change” [(14), p. 119–120, italics mine].

And this:

“Jokingly it has been said that psychomotor therapy is the psychoanalysis of the body. It releases material from the *subconscious,* as in classic analysis, but in a different way. This occurs as an integral part of the physical changing process” [(14), p. 120, italics mine].

Returning now to Anne's case: Since individualization is essential in any professional activity, a few remarks on how her background, condition, and problems relate to characteristics of the NPMP treatment need to be added.

*About the patient*: Her background story, current ailments and reactions as well as the combination of her bodily and emotional blockage are viewed as expressing protection, a need to keep unpleasant memories and feelings at bay. That she had “odd visions” which the psychiatrist interpreted as a sign of a psychotic state only strengthens the impression of someone suffering severe strain.

*About the treatment*: The treatment can be characterized as stabilizing and supportive. It was important that the patient herself was active and participating. Active exercises without hands-on assistance were therefore central in the therapy; the point was to let the patient control the situation and it was crucial to begin by helping her establish a sense of mastery—to let her realize that she held the reins.

The treatment was thereafter broadened to include massage—touch—beginning at the patient's feet and later including the rest of the body. The precise order is important here: first the feet which to most people is a rather neutral part of their body, and finally the neck/throat/face which are associated with feelings, proximity, and intimacy. Especially the face. But the feet are themselves vital here. They have a key function with respect to stability and change as well as to the spring in one's step and the strength in one's stride.

#### 3.3.1. Further reflections

The therapeutic approach reported on above contrasts sharply with NPMP as a loosening up and liberating form of therapy. What could be the rationale for the choice of a supporting form of therapy in this case?

When it became evident that Anne disliked touch, the therapist interpreted this as a message saying, “please stay away”. In this situation, the appropriate thing for the therapist to do was to respect the patient's experiences and limits in order not to evoke painful memories, i.e., to avoid touch altogether. However, when the typical features of NPMP practice—the on-going bodily interaction between the two parties, including much hands-on techniques—had to be dropped, the therapist lost an important source of information (on changes in tensions, breathing, skin temperature, perspiration, etc.). She also lost a central communicative channel. Now, she had to rely solely on her sight and hearing. The challenge was to try to help Anne without touching her as a start and see what happens.

Since Anne seemed to have alienated herself from her body, it was paramount to assist her in establishing better grounding—bodily anchoring—as the first step towards developing body contact and confidence. To this end, the physiotherapist suggested exercises and a variety of movements.

There is not much to be learned in the text about how the physiotherapist proceeded, how she worked with Anne's movements, how the two of them interacted, which exercises were selected, how movements were individualized, and so on. However, since the physiotherapist had a psychomotor background, we may surmise that she was determined to choose exercises and movements that would give Anne new and positive bodily experiences. In turn, these experiences would hopefully help her overcome bodily habits and manners entrenched since early childhood, thus stimulating a feeling of mastery and control. Based on Anne's words in the interview, the physiotherapist's approach proved to be helpful and effective for her.

We can also assume that the physiotherapist was fully aware of how demanding the process would be for the patient since changing habits and movement patterns is not merely a question of changing one's motor capacity; it involves one as a person. What is required for the therapy to succeed, is the patient's investment and active participation over time.

In recent decades it has been well documented that people with a traumatic past (be it violence, abuse, or neglect) often establish a distance to their body as an integrated part of a general defense against negative, overwhelming feelings (41–43). Many of these persons function precisely *by way of* their defense. It is therefore essential, in such cases, to abstain from a liberating and loosening up approach, and to begin instead with a therapy that stabilizes and supports—reserving more relaxing interventions for later.

Given the increasing public and health care attention to traumatic experiences, the relevance of Anne's case for physiotherapists in general, not only for psychomotor therapists, should be obvious. The sheer number of persons who have experienced some form of violation exceeds what most people would have thought is the case. This, too, underscores the relevance of this case: wherever physiotherapists work, they are likely to meet patients who struggle with the impacts of a difficult and painful past.

### 3.4. Examination—Peter

Four excerpts from the clinical part of the examination in one and the same encounter are presented in the following (22). They are typical for how psychomotor physiotherapists examine patients. Two excerpts are from sequences in which the physiotherapist observes the patient's positions and movements (i and ii) and two are from palpation (iii and iv). For my present purposes, the passages are partly abbreviated, partly expanded.

Since the aim of the NPMP examination is to obtain information about the body's flexibility and versatility, the clinical part of it is pivotal. Even so, it may be helpful to begin with a summary of the history-taking because this talk sets the tone for the rest of the encounter.

The patient, Peter, who lived with his girlfriend (no children), was referred to psychomotor physiotherapy for muscle tensions. Both patient and therapist were men in their 40s and the examination took place in private practice in the primary health care.

#### 3.4.1. History-taking

During history-taking, Peter said that he had suffered from asthma and eczema and that he had some trouble with his stomach. He stressed, however, that his muscle tensions, particularly in the neck, were his main problem.

Then his work situation came up. He had two jobs, and a heavy workload. During the day he worked as a computer specialist, and in the evenings, he helped rehabilitate young drug abusers. Peter expressed explicitly that he thought there was a connection between his muscle tensions and his job situation [(22), p. 172].

As to Peter's asthma and eczema, the physiotherapist asked him how he experienced these disorders. Peter answered that he hardly thought about his eczema. But he got easily congested and he was sensitive to cold. As a child, he limited his physical activity, due to his breathing problems, never playing football with his classmates. As an adult, he became “more active”, periodically “getting in good shape”, being less bothered by his asthma. But “I am still a little handicapped as far as physical activity goes”.

The history-taking ended with the physiotherapist asking the patient if there were anything else he would like to talk about, anything about his situation. Peter repeated that he pushed his body, that he had “to keep on the move”, adding that, “maybe I can start some process that would let me live a quieter life and manage to use my energy more optimally” [(22), p. 174].

The physiotherapist asked no more questions, nor did he say anything about his assessments. He then moved on to the clinical examination.

As this brief summary shows, the history taking was concentrated on the patient's bodily symptoms, experiences and disorders, and how he functioned in his daily life. It also shows that the patient was active right from the start. The physiotherapist encouraged Peter's participation by listening and asking questions that were directly related to his contributions. His version was, in short, given priority. The physiotherapist limited himself to repetitions, reflections, and more probing questions.

#### 3.4.2. Excerpts from the clinical examination

##### 3.4.2.1. Habitual patterns and quality of movement

The physiotherapist pushes a stool towards the patient and asks him to sit on it the way he usually sits. Peter sits down, hunches over and says, “This is how I sit” [(22), p. 175]. The physiotherapist immediately learns not only how Peter habitually sits, but also that he knows it. Then, using his hands and voice the physiotherapist asks Peter to sit this way and the other, letting him sense how sitting in different positions feels.

Still sitting, Peter is asked to open his legs slightly, moving his knees lightly back and forth. The physiotherapist assists him by gently pushing on his thighs. Peter smiles a little, looking somewhat embarrassed.

Next, Peter is asked to bend forwards. Again, the physiotherapist assists his movement, asking him to relax his neck and arms. The movement is repeated several times, Peter being encouraged to feel the play of gravity, to take note of his movements when he holds back and when he lets himself be moved. The two of them also work on movements in the opposite direction, from being bent forward to sitting up straight.

All the while the physiotherapist continues his encouragement using words and his own body, especially his hands. He comments on what he observes and helps support the movements. Thus, Peter gets the opportunity to become aware of what he does.

##### 3.4.2.2. The ability to isolate muscle contraction and to relax

This sequence begins with Peter lying on his back. The physiotherapist asks him to tense one thigh (straightening his knee). Peter makes a tremendous effort, tensing muscles in several parts of his body. The physiotherapist asks him to “use less energy”. They repeat the exercise a few times before the physiotherapist asks the patient if he is aware of what he is doing. The patient laughs a little and says he is “not sure what he feels” [(22), p. 176].

The physiotherapist strokes Peter's thigh a few times, slowly, using his flat hand. Then he alternates between massaging quadriceps and quick, stimulating grips above *m. vastus medialis*, right above the kneecap, asking Peter to stretch his knee.

The talk continues:

“*T:* It looks to me like you’re tensing your seat too

*P*: Yes

*T*: We’ll get back to this sort of thing later. You could say that … the purpose of this is to get an idea of whether you can manage to tense the part down by the knee and not tense up in several places. Try it again and see if you can do it without using much energy” [(22), p. 176].

This functional test reveals that Peter overdoes it, and he is not entirely aware of exactly what he does in terms of bodily response.

##### 3.4.2.3. Palpation and conversation on the therapist's initiative

In this sequence Peter lies on his stomach. The physiotherapist keeps one hand on Peter's shoulder, placing his other hand on the small of Peter's back, pushing towards his feet, reducing his lumbar lordosis. Importantly, this stimulates a more basal respiration.

The physiotherapist then strokes Peter's thorax and back with long, calm movements, intermittently performing certain loosening grips in Peter's shoulder area and his lumbar region. Little by little, Peter's breathing turns a little more basal.

Then the following conversation takes place:

“*T:* You said your stomach reacts. Can you feel it in your stomach if you have a bad conscience?

*P:* Yes, I have digestion problems, fairly often

*T:* Can you describe them?

*P:* Sometimes my stools are hard, sometimes loose. I’m a big eater, even though I’m fairly thin. My digestion as a whole isn’t very stable, or even okay, I don’t think.

*T:* Are you troubled by gas problems too?

*P:* Yes, lots of gas” [(22), p. 176].

The physiotherapist refers back to what Peter had said during history-taking and tries to expand on that information. It tells the patient that the physiotherapist has noted what he has said and attaches importance to it. The physiotherapist does this several times during the examination.

##### 3.4.2.4. Palpation and conversation on the patient's initiative

In this last sequence, Peter lies on his back on the treatment bench, the physiotherapist sitting behind him, cradling Peter's head in his hands while palpating his throat, jaw, and face.

The following exchange takes place:

“*P:* When things are really bad, I have a habit of tensing my jaw something awful

*T:* Can you show me how you do that? (P shows how he tenses his jaw)

*T:* Does anything else happen when you clench your jaws together that way?

*P:* Yes, as a rule, when I wake up like that I’m pretty tensed up” [(22), p. 177].

The physiotherapist now learned that Peter is aware of the connection between jaw tension and his emotional state. He also learns that Peter *recognises* his bodily reaction patterns so that he can demonstrate them, i.e., he is not out of touch with them.

The physiotherapist continues palpating and asks Peter to stretch his lower jaw forwards. The physiotherapist assists him by pushing lightly under his chin. Then he asks Peter to move his jaw, flexibly from side to side, wiggling. Peter immediately stretches his jaw forcefully left and right as far as it will go. The physiotherapist intervenes by asking him to use as little energy as possible, to make tiny, effortless movements. Peter clearly finds this difficult and the expected liberating effect on his breathing fails to manifest.

The physiotherapist then asks Peter to open his mouth widely. Again, Peter uses much force, tilting his head backwards while holding his breath. The physiotherapist encourages him to restrict his movements to his jaw only. Holding Peter's head in his hands, the physiotherapist asks him to open his mouth once again.

While holding Peter's head, the physiotherapist pulls it towards his own body, effectively giving Peter's neck some traction. Asking him once more to open his mouth, Peter observes that he is unable to do so without activating his neck. The physiotherapist replies that they will return to this in due course.

##### 3.4.2.5. Closing conversation

The closing conversation, after the patient got dressed and while the two sat facing each other, began by the physiotherapist asking Peter how he had experienced the examination. He answered that he did not really “understand” much of what the examination involved but added that he had become more conscious of his tensions and his difficulties in relaxing which he interpreted as indicating his need for professional help.

The physiotherapist repeated that he thought Peter would benefit from NPMP therapy, saying, “We’ll have to see how far we get. We’ll try a few times and see how it goes … it's hard to say how quickly you can expect to see any change” [(22), p. 178].

As regards his own observations, interpretations and evaluation of the patient's health problems, he did not say much. About the therapy he pointed out that the patient's own participation is essential, that the treatment is a “joint project” [(22), p. 178], the outcome of which is impossible to predict.

#### 3.4.3. Comments

As the four sequences above demonstrate, the physiotherapist used touch in a variety of ways. He tried different grips and movements related to posture, muscles, respiration, and several times, adjusting his approach slightly as the patient responded. Thus, Peter was given a chance to try different movements, to do things in different ways, to *feel* the differences, and to learn something about himself.

The way Peter's physiotherapist used touch accords with NPMP principles. Importantly, and in contrast to traditional diagnostics, psychomotor physiotherapists actively intervene with words, hands, and body throughout the clinical examination. The trial treatment in NPMP is not about performing tests supervised by a detached observer. It is a social situation involving exploration, with the physiotherapist as a participant observer.

Thus, the physiotherapist followed up on what he heard about tension and being stressed by perceiving through his own fingers and body how what Peter said corresponded to what his body conveyed. Peter's experiences were not isolated, verbal “inner” matters that belonged to the history-taking. They were bodily experiences *and* expressions that could be observed *and* talked about throughout the examination.

Therapy seeking is always preceded by problems of some kind and certain interpretations of experience. What patients look for is the acceptance and validation of their experiences by a professional, a means to account reasonably for their feeling that something is wrong. Not experts themselves, they want explanations and strategies to deal with the situation.

Although this encounter did not bring the patient much factual information, reasons and explanations, Peter did get something else, something not usually associated with knowledge: the examination gave him new bodily experiences and hence a chance to develop a form of bodily awareness and acknowledgement.

Having concluded his examination, the physiotherapist's proposal for treatment was in keeping with his understanding of the problem and his rather imprecise definition of it. He had clearly got the impressions he needed to go on with treatment, and he stated that the patient could “benefit” from the treatment [(22), p. 178].

During the encounter, Peter and his physiotherapist discussed only fleetingly the eczema and breathing difficulties that Peter reported, and his stomach trouble became a topic only when the therapist introduced it while he was palpating Peter's back (sequence 3.4.2.3). But it was dropped quickly. The physiotherapist said very little even about Peter's main problems.

In my post-encounter interview with the physiotherapist, I questioned him about this, being somewhat surprised. “Yes, why didn’t I say more?”, he asked himself pensively [(22), p. 182]. One thing was that he had treated several other patients who were employed at Peter's workplace and his impression was that the atmosphere there encouraged psychologizing and intellectualization. Then he added that he felt Peter's understanding would improve with time and bodily experience. In his view, Peter was “good at expressing himself; he had an “intellectual understanding”, but he wondered how much he had actually understood. What Peter needed, he repeated, was “bodily experience” [(22), p. 182]. Thus, the physiotherapist presented himself clearly as a physiotherapist, a professional provider primarily concerned with patients’ bodily function.

## 4. Discussion

In what follows several interrelated issues pertaining to the NPMP practice reported on above will be addressed. Although the focus is on this tradition, the aim is to highlight issues relevant to clinical work in physiotherapy generally.

It should be evident already that the three physiotherapists adapted their practice to the individual patient and that touch and hands-on methods were crucial in how they sought to individualize their therapy, their main concern being to strike a balance between a liberating, loosening-up approach, and a stabilizing, supporting one. Hopefully, it has also become clear that the therapists' actions were informed by a view of the body as a historical phenomenon in accordance with central tenets in NPMP. This must be stressed, because not only is it pivotal for how the physiotherapists carried out their clinical tasks, it is also essential for the patients' potential for participation and learning.

Certain aspects of the way the physiotherapists used touch and movements will be taken up again presently, given that both are at the heart of physiotherapy in general, just as changes in bodily habits remain an essential if challenging professional aim because one's ingrained bodily habits are part of who one is; one's identity is at stake. A discussion that has been going on in recent years about the conversational content in psychomotor encounters will also be addressed. But first to certain parallels between NPMP and phenomenology as a philosophy of the body.

### 4.1. Phenomenology—a source of inspiration

Although Braatøy was widely read, it is highly unlikely that he was acquainted with the work in philosophy that became foundational for later thinking on the body, *Phenomenology of Perception* (PP) by Maurice Merleau-Ponty (26). Yet the NPMP that Braatøy and Bülow-Hansen developed is so “in tune” with fundamental insights in PP that one can mine the book for parallels. There is nothing in NPMP that runs counter to the fundamentals of phenomenology and were one to imagine a physiotherapy deriving directly from phenomenology it would have much in common with NPMP.

It is not suggested that NPMP approaches the depth and complexity of phenomenology. But more can be done to strengthen the theoretical foundations of NPMP by drawing on phenomenological perspectives.

To begin with, and as stressed repeatedly, in NPMP the body is understood as a historical site—it is the repository of the person's past, harbouring recollections of its own kind, often inaccessible to the conscious mind. This tallies well with the phenomenological understanding that as human beings we cannot ever be detached from our corporeality [(26), p. 90–97].

My body is qualitatively different from any other object in the world because it is not something I simply have, it is something I also *am*: I *exist* as body. Body and self are permanently one and the same phenomenon. The double character of the body means that I am always “intertwined”: a visible-seer, a tangible-toucher, etc. [(26), p. 90–97]. The subject-status is, however, primary in the sense that it is as an experiencing and expressive embodied being I go around doing my things, and I am still an experiencing body when gravity pulls on me as a material phenomenon.

This two-sidedness also means that the self can objectify itself *qua* body—it can assess whether a particular physical task is feasible, for example. But by and large, as we go about daily routines (walking down the street, drinking a glass of water), we are spared of having to apply our limbs consciously, just as we don't have to remind ourselves that gravity pulls.

The concept of incarnate subjectivity has clear parallels in NPMP understandings. In all the examples above, the therapists face patients whose past experiences have solidified—materially, as it were, in their corporal being—in muscle tensions, hampered movements and reduced function.

This means that phenomenology, especially as it was formulated by Merleau-Ponty—the theorist who most systematically grounds human subjectivity in the body (27–29)—can serve as inspiration not only for improving clinical practice, but also for an improved theoretical underpinning of NPMP. Moreover, phenomenology is an excellent point of departure for critiquing kinds of physiotherapy that expressly disregard the body's double character—practices that concentrate exclusively on the body's quantitatively calculable properties. Such physiotherapy tends to separate the physical body from the self, confounding the person's understanding of what he or she *is*, simultaneously encouraging an objectifying stance towards the body which becomes a vehicle for measurable accomplishments. In today's body-oriented climate this easily leads patients to cultivate an observer-relation to their own body (30, 44).

### 4.2. NPMP, neuroscience, neurology, and phenomenology

This is not to say that the body's “sheer materiality” is to be neglected. On the contrary, advances in neurology and the neurosciences over the past few decades have reinforced classical phenomenological insights just as they have confirmed fundamental NPMP premises. Phenomenology and NPMP both go beyond the silent body, a machine analogue, and the speaking mind, detached from any form of materiality. Both traditions *unite* what for centuries have been separated: body parts and body systems, action and perception, movement and communication, experience and cognition, although there are significant differences between them, particularly with respect to philosophical depth and explanatory consistency.

#### 4.2.1. Neuroscience

The main conclusion from neuroscience research is the overwhelming evidence for bodily interaction. Previously, different body systems, often analysed separately are now known to be highly interacting. These findings have resulted in novel terms such as psychoneuro-immunology (PNI) and psycho-neuro-endocrino-immunology (PNEI), the point being that interaction takes place in complex ways, depending on a host of conditions pertaining to the persons involved as well as to their environment. In other words, human experience *as such* has both bodily and psychosocial aspects that are inextricably related (34–36, 43–46).

Through innumerable studies during the last decades, it is documented how stressful events and long-standing strain of various kinds (war, hunger, neglect, abuse, relational problems, lack of sleep, lack of physical activity etc.) lead to physiological dysregulation which in turn enhances disease susceptibility (cf. the concept “allostatic overload”) (43, 45–49).

Likewise, and from its very inception, NPMP has viewed the body-subject as a functional and interactive unity, a juncture between nature and culture. This has profound therapeutic implications. For example, psychomotor physiotherapists take it for granted that their approach has effects on more than muscles, tendons, and joints. They also take it for granted that experiences remain with us as part of our embodiment, and that they can be channelled and manifest in multiple ways.

Turning to the patients reported on above, the effects of long-standing stress seem obvious: In addition to muscle tensions, Katrine suffered from headache/migraine, Anne from nausea, Peter from eczema, asthma, abdominal trouble. What those three cases also indicate, but indirectly, is the importance of the *longue durée* of NPMP therapy.

Except for the first few years when the approach was in its rather experimental infancy, psychomotor physiotherapists have always emphasized the importance of gradual change. That is to say, one has sought to avoid quick fixes aiming to produce catharsis-like effects (11–15). The immediate reason for this is to preclude aggravation of the symptoms and problems that brought the patient to seek expert help in the first place. An equally significant reason is the concern for the body as a whole—the crux of the matter is to prevent dysfunction in other body systems (11–15).

In NPMP one has been particularly attentive to autonomic dysfunction, and to whether the treatment results in too much relaxation and liberation of the respiration, i.e., more than the patient can tolerate emotionally, and thereby risk provoking reactions such as palpitation, sleep disturbances, problems with temperature regulation, etc. In NPMP this is known as “body systems switching”, something Braatøy discussed 75 years ago [(11), p. 269–339].

#### 4.2.2. Neurology—proprioception—body schema and body image

Findings in neurology are no less central. This pertains especially to certain features of proprioception that are highly relevant to physiotherapy.

Often referred to as the “sixth sense”, proprioception allows me to know, without needing to ascertain it, that I have both feet on the ground, that my legs are crossed or that when I run, only one of my feet touches the ground at the same time. This takes place “automatically” because receptors in the musculoskeletal system (together with receptors in the skin and the inner ear) inform the central nervous system about how the body is positioned and the way it moves (32). In other words, the body itself provides the systemic input, “liberating” the self from the task of deliberately monitoring the sensorimotor processes involved in standing, sitting, walking and running.

But there is more, and arguably equally important from a physiotherapeutic perspective: If one's proprioceptive feedback disappears, one loses control not only of posture and movements, but also of a certain feeling of self, a sense of integrity and reality (28, 29, 32, 33, 50–52). Testimonies by patients who have lost their kinaesthetic or proprioceptive sensation alert us to the often-neglected function of proprioception: the natural, unreflected self-feeling. This should remind us of the therapeutic potentials in promoting movement when we aim to boost people's sense of anchorage. These patients had to compensate for their loss by visual guidance and cognitive intervention, but despite continuously straining to concentrate they succeeded only partly in regaining control over posture and movements. However, their weird feeling of being lost, disembodied, and alienated remained.

The lesson to learn from this is, again, that we should ascribe high value to variation in positions, movements, and activities as a means for supporting a bodily grounding—a feeling of being a distinct “I”—the very locus of agency.

It is useful at this point to invoke the conceptual distinction between body schema and body image since much literature is confusing with respect to these concepts (28, 29).

Body schema is the English translation of Merleau-Ponty's concept *schema corporel*. He was concerned to grasp the essence of how human beings are practical agents in the world. He portrays the body as a “knower”—we have “a grip” on the world before we come to know it verbally and consciously. Knowledge is “in the hands”, he writes, it is “the body that understands” [(26), p. 137–47]. Most of the time our attention is directed away from the body and toward the environment or some project we are undertaking. As Merleau-Ponty puts it, “Consciousness is not in the first place “I think”, but “I can’” [(26), p. 137]. According to his philosophy, one cannot be a subject without a body. The subject experiences the world through the body, one's access to the world is bodily: my body is “the pivot of the world”, that “*by which* there are objects”, he writes [(26), p. 82, 92].

According to Gallagher and Zahavi, the “concept of body schema involves two aspects: (1) the close-to-automatic system of processes that constantly regulates posture and movement to serve intentional actions and (2) our pre-reflective and non-objectifying body awareness” [(29), p. 165]. The body image, in contrast, “is composed of a system of experiences, attitudes, and beliefs where the object of such intentional states is one's own body” [(29), p. 165].

A central difference, then, is that the body schema reflects the body's practical adjustment and attunement to the environment in an all but automatic way, while the body image refers to the conscious or reflective activity of one's own body.

While conceptually distinct—although “the distinction in question is not that easy to make” [(28), p. 24]—these two systems interact in various ways. For example, the unattended flow of movements that characterizes most actions in daily life may be disturbed if we begin to think how we move (type, play an instrument). In contrast, it is useful to pay attention to our (own) body and focus on details in the way we move when we learn new skills (cycling, riding, skiing), at least initially, before we become competent agents and can “forget” ourselves and direct our attention outwards.

This is precisely what happens in NPMP when physiotherapists help patients to first become aware of their habits and bodily manners and then to turn dysfunctional patterns into more appropriate ones. As it was put 30 years ago: “The thread running through psychomotor therapy is the experience of the body and of experiencing through the body” [(14), p. 71]. What the therapists in the three cases above did was to appeal to the patients' awareness and consciousness through exploring movements, using touch and talk. Learning through concrete bodily experiences combined with reflection is a key feature in NPMP.

#### 4.2.3. Touch, movement, talk

From a phenomenological perspective, human beings are incarnate social subjects and bodily messages are exchanged continuously whenever people meet in so-called “face-to-face” encounters (more aptly: “inter-corporeity” encounters). As embodied, historical beings we cannot, following Merleau-Ponty, *not* communicate; we are doomed to be accessible for one another. We are visible seers, as Goffman and other micro-sociologists and interactionists have made abundantly clear (31, 38).

And we are doomed like this from the very beginning. It is the core of sociality. This is exactly what theorists such as Trevarthen (53), Mead (54), Bourdieu (55–57), Connerton (58), and Crossley (30, 59) help us to understand. As Bourdieu puts it: “The body is in the social world, but the social world is in the body” [(57), p. 15]. In this view, socialization, both primary and secondary (i.e., first in the family, and later in the wider societal context) is inevitably an embodied affair. We grow into norms and values not only mentally, but also—and simultaneously—as experiencing and expressive bodily beings. This perspective is highly relevant for our profession: It means that the social is directly expressed in the way we move, and in the activities we engage in.

Movements then, are not to be conceived of in isolation, since they are an integrated element of a greater whole, including perception and appreciation, cf. Bourdieu's concept “habitus” (55–57). But physiotherapists know that habituated movements can be dysfunctional. People are sometimes unwitting victims of their habits and their past. As Crossley reminds us, “old habits, as the saying goes, die hard” [(30), p. 129], and people “will take many habits, acquired in infancy, to their grave [(30), p. 130]]. This is so because in a fundamental way our habits are who we are. To embark on changing one's habits is therefore to embark on changing one's identity. Still, for a physiotherapist to refrain from helping patients shed injurious habits and dysfunctional movements can obviously be a disservice to them.

Again, insights from phenomenology may assist the physiotherapist in grasping the nature of the patient's problem and again, the starting point is the body-I as “*a knower*”. This body-subject is always *intentional*: it is directed outwards. While Husserl was primarily concerned with intentionality as a feature of consciousness, Merleau-Ponty links intentionality to our embodied existence. For him, *motility* is “basic intentionality” [(26), p. 137], “to move one's body is to aim at things through it” [(26), p. 139]. The lived body is conceived of as an embodied consciousness with a “from—to structure” that simultaneously engages, and is engaged in, the surrounding world. For humans, physical space therefore becomes oriented social space.

Merleau-Ponty elucidates how action and perception are a unity, they are “two facets of the same act” [(26), p. 205]. We respond through movements to the requirements of our surroundings, and things appear to us depending on our bodily capacities. Scaling a cliff may be easy for a trained mountaineer whereas climbing a staircase may seem impossible for a hemiplegic. There is a mutuality between what we are capable of doing and the manner in which we apprehend the world [see also (30, 44, 59)], a reciprocity between what we master and how the world appears to us that is of utmost importance for physiotherapists to understand.

In NPMP, the primary interest lies in the *quality* of movements. Focus is on flexibility and versatility, with emphasis on nuances in bodily bearing in different positions and in *how* persons move. The concern is to stimulate the patient's participation and involvement, opening up new areas of experience, expanding one's movement repertoire—not only of using “one's body”, but using *oneself*. Or to be more precise: of being-in-the-world, to employ a phenomenological expression. Attention is paid to helping patients establish bodily contact and confidence, and thereby to rely on their agency. What is more: with new experiences, expanded repertoire and increased confidence, the patients' world changes.

As emphasized above, psychomotor physiotherapists are concerned with *gradual* change. This is why treatment includes frequent repetition of exercises and movements that patients master as well as gradually introduced movements that challenge dysfunctional habits and bodily manners. The kind of change these physiotherapists are after is achievable given the ability of the human central nervous system to alter and modify its structure and function (32). Understanding this plasticity has opened for a more optimistic view of the possibilities for improvement and has contributed to a greater emphasis on rehabilitation generally. It is now well documented that plasticity is use-dependent and closely linked to learning.

As Brodal writes:

“The vast number of neurons and plasticity of the human brain enable learning of an almost infinite repertoire of voluntary movement. Also, these features ensure great flexibility in how motor tasks are solved” [(32), p. 279].

Interestingly, he also stresses the difference between humans and animals:

“Most animals are highly specialized for a limited number of motor tasks, controlled by stereotype motor programs that develop according to fixed pattern. Humans in contrast can solve tasks in different ways, and we can continuously adapt to novel challenges” [(32), p. 279].

This difference of movement patterns and solutions between humans and animals is of vital relevance in physiotherapy. It is reasonable to see it as an invitation to make active and conscious use of the potentials inherent in human nature.

Another key difference is language. That the strictly physical aspects of exercise and movement have many benefits is known by all, but it is argued here that the *combination* of body work and talk has great therapeutic potential. To put it differently, bodily and verbal forms of communication—or “dialogues”—can complement each other in a fundamental way when verbal and bodily information are no longer understood as categorically different, belonging to the separate worlds of “matter” and “mind”.

It is pertinent now to return to the statement by Katrine's physiotherapist above, that physiotherapists are not to function as psychologists. What she implies is that to talk with patients about their problems in life is what psychologists do; such talk belongs to their domain of responsibility. It is worth noting that lately, psychomotor physiotherapists have been discussing the content and importance of the conversation in clinical encounters, a point I now turn to.

In the psychomotor physiotherapy tradition, it is emphasized that we obtain valuable information from patients while we examine and treat them. Patients often relate something important about themselves and their life in these situations and they tend to do so more spontaneously than when they engage in conversation, sitting face-to-face. But the psychomotor physiotherapist was always encouraged to be a good *listener*, not a great conversation partner. To put it in terms that most NPMP practitioners will recognize: they were supposed to function as the patient's “wailing wall” (12–15). To engage in conversation beyond this was considered to encroach on the physicians' (and, in the early years, the psychiatrists') domains.

Nowadays this division of labour is questioned since it is clearly not in keeping with fundamental NPMP precepts. There are those who think that conversations should be given greater emphasis, stressing that this should not be taken to mean that physiotherapists should function as psychotherapists or become “verbal therapists”. This would obviously require a different education.

Returning once again to the cases above: we saw how in retrospect Peter's physiotherapist wondered why he had said so little while Katrine's therapist intervened, noting Katrine's eyes dimming she immediately remarked on it. Now, an important difference between the two cases is that the encounter involving Peter was his first: the examination. For all we know, his physiotherapist was to be more forward as the therapy proceeded. Katrine was in her fifth session, and it is reasonable to assume that she had developed trust in her therapist, allowing herself to “let go”. But still, by confronting her so directly, the physiotherapist took a great leap from what for so long has been the NPMP attitude.

The new here—the change that has taken place—concerns an about-turn in *what* psychomotor physiotherapists “permit” as topics of conversation, and in *how* they follow up verbal and bodily expressions. Psychomotor physiotherapists have become more active in asking how the patient is doing, they make note of what patients say and what they don't say, and they initiate talk about topics the patients themselves do not introduce. These physiotherapists have become more offensive, and their increased boldness is in my view all for the better.

### 4.3. Methodological considerations

In preparing for this article, my first priority was to select rich, thick descriptions of actual clinical practice in Norwegian psychomotor physiotherapy (NPMP). Having sifted through textbooks, a host of journal articles and post-graduate theses, it became clear that while textbooks provide technical how-to instructions on touch and grips, and practically all articles and theses on NPMP mention touch as a more or less integral part of clinical practice, the very *significance* of touch is largely passed over. So, when Bjorbækmo and Mengshoel (60) state that touch is central to much clinical practice in physiotherapy generally but largely absent from physiotherapy-related research, it can be added that NPMP is a tradition in which touch is a central element both in practice and in texts, but very few authors manage to combine concrete descriptions of touch with relevant and consistent theoretical considerations.

Equally important was to select texts that demonstrated how physiotherapists approached patients' problems and where the therapeutic potential of touch was discernible. The three texts reanalyzed in this article were selected for the following reasons:

The first text (20) came to mind immediately because it draws attention to a well-known challenge in NPMP sessions: a patient's release of pent-up emotions—in this case, in the form of tears—after having been touched. It also introduces to the reader the significance of talk in a clinical setting, or rather, the therapeutic potential in combining talk and touch.

The second text (21) was selected because it brings out in considerable detail how problematic touch can be (the patient in question abhorred being touched). It also shows the vast therapeutic potential of the gradual, almost imperceptible introduction of touch. It deals with a patient diagnosed with psychosis and should be highly relevant given the current concern in the health sector with the increase in mental problems.

The third text (22) was selected for two reasons: it is a detailed account of an NPMP *examination* (or trial treatment). It is thus of intrinsic interest since patients are normally referred to physiotherapy with vague diagnoses such as myalgia—or, as has been possible in Norway since 2018, patients arrive at the physiotherapy clinic without referral. Physiotherapists must therefore find out what the problem is before they can decide what to do. The other reason is that in this text, written nearly 30 years ago, the omnipresence of touch in the examination excerpts is striking, something the article (22) does not draw attention to.

Since these three texts are based on different kinds of material, though all of them deal with interactions between patients and NPMP therapists, a comment is in order. In the first case (on Katrine), the first author describes her own clinical experience (20), the second case (on Anne) is based on a retrospective interview between a patient and a psychologist about the patient's experiences with a combination of psychotherapy and psychomotor therapy (21), and the third text (on Peter) is based on my own observations of a clinical encounter, supplemented by an interview of the NPMP therapist (22).

About this third article there is little to add except that the focus then was on the differences between examinations in medicine and physiotherapy [see (22), p. 167]. Rereading it now revealed that much more could have been said about touch and hands-on means, and how these means were an integral part of the diagnostic task. The reason the text on Anne (21) stood out was that since touch repulsed the patient, touch was counterindicated. The therapy progressed by way of simple exercises and light, cautious handling, beginning with the patient's feet. I have no personal knowledge of either Anne or her therapists. Regarding the excerpts on Katrine (20), it should be pointed out that there is a possible shortcoming in that the text is based on the physiotherapist's recollections. Even if they are based on notes taken for the patient record there are of course certain limitations with texts that rely on a clinician's memory. No one remembers everything from a certain event and setting, and much clinical practice is carried out more or less intuitively and unconsciously, based on experience and established habitual skills. I agreed to serve as the second author because I knew the first author well, and because she had several years of experience with traumatized patients. Based on my own knowledge of NPMP as long-term therapist, lecturer, and researcher, I am confident that the descriptions of practice in the article (20) is typical for approaches in NPMP. Still, and as indicated above, the therapist was sufficiently self-assured in the situation to probe verbally and bodily further than most NPMP therapists are accustomed to do. From this, a lesson can be learnt.

### 4.4. Final remarks

Together, these three cases cast new light on how touch and talk are complementary modes of communication. They show the importance of comprehending communication as an integral part of therapists' carrying out their diagnostic and therapeutic tasks. Put differently, communication is not something that happens in addition to the “real” professional work (17, 24, 30, 31, 37, 38, 59).

Moreover, the analysis demonstrates how perspectives from phenomenology and the social sciences, together with new knowledge from the neurosciences represent potentials for anchoring physiotherapy practice in an extended framework. This way, the current understanding of the significance of touch both in NPMP and in physiotherapy generally is augmented.

As noted above, in Norway, patients' access to physiotherapy without a physician's referral has been possible only since 2018. In the current health-conscious climate, physiotherapy can thus become a consumer good for those who have the time and resources to seek it out on “a regular, prophylactic basis, and at their own expense”, as Moffatt and Kerry put it [(8), p. 184]. This perspective is certainly worth pursuing even if legislation varies greatly from one nation to the next as does the availability of physiotherapy and its institutional organization.

Still, there is undoubtedly much to be gained by beginning with the obvious: physiotherapy is a practical professional activity. Its legitimacy derives from physiotherapists' actions and the consequences of these actions. In my view, it follows that focus on real-life clinical situations is of utmost importance. In this article, numerous excerpts from actual practice have been presented, focusing on the combination of touch, movement, and talk. The rather detailed presentation of three very different encounters has made it possible to demonstrate how I deal with the material and for readers to evaluate how I proceed from description to analysis. I also hope that I have been able to make a case for more research in the future on actual clinical practice—not only on NPMP but on physiotherapeutic sessions in general.

## Data Availability

The original contributions presented in the study are included in the article/Supplementary Material, further inquiries can be directed to the corresponding author/s.
